# Circular RNA Circ_0067934 Attenuates Ferroptosis of Thyroid Cancer Cells by miR-545-3p/SLC7A11 Signaling

**DOI:** 10.3389/fendo.2021.670031

**Published:** 2021-07-05

**Authors:** Hui-Hui Wang, Jia-Ni Ma, Xiao-Rong Zhan

**Affiliations:** ^1^ Department of Endocrinology, The First Affiliated Hospital of Harbin Medical University, Harbin, China; ^2^ Department of Endocrinology, Qiqihar First Hospital, Qiqihar, China; ^3^ Affiliated Qiqihar Hospital, Southern Medical University, Qiqihar, China

**Keywords:** thyroid cancer, ferroptosis, circ_0067934, miR-545-3p, SLC7A11

## Abstract

Ferroptosis is an emerging programmed cell death distinguished from apoptosis and autophagy and plays essential roles in tumorigenesis. Thyroid cancer is a prevalent endocrine tumor, but the molecular mechanism of ferroptosis during thyroid cancer development remains unclear. Here, we identified the critical function of circular RNA circ_0067934 in repressing ferroptosis of thyroid cancer cells. Our data showed that the ferroptosis activator erastin decreased thyroid cancer cell viabilities, while the circ_0067934 shRNA further attenuated erastin-inhibited cell viabilities. The silencing of circ_0067934 enhanced the levels of ferroptosis-related markers, including Fe^2+^, iron, and ROS in the cells. The knockdown of circ_0067934 induced thyroid cancer cell apoptosis and repressed thyroid cancer cell proliferation *in vitro* and *in vivo*. Circ_0067934 upregulated the expression of the ferroptosis-negative regulator SLC7A11 by sponging and inhibiting miR-545-3p in thyroid cancer cells. The overexpression of SLC7A11 or the inhibitor of miR-545-3p reversed circ_0067934 silencing-regulated thyroid cancer cell proliferation. Therefore, we concluded that Circ_0067934 attenuated ferroptosis of thyroid cancer cells by miR-545-3p/SLC7A11 signaling. Circ_0067934 may serve as a potential therapeutic target by regulating ferroptosis for the treatment of thyroid cancer.

## Introduction

Thyroid cancer remains the most frequent endocrine system tumor with an increasing rate in multiple regions and countries (1, 2). Thyroid cancer can be histologically classified as anaplastic thyroid cancer (ATC), medullary thyroid cancer (MTC), follicular thyroid cancer (FTC), and papillary thyroid cancer (PTC) (3). Though PTC presents a favorable prognosis, there are yet 10%–20% of thyroid cancer patients have the features of recurrence, drug resistance, distant metastasis, and local invasion, leading to a low survival time (4, 5). Ferroptosis is a complicated process of programmed cell death distinct from apoptosis and autophagy (6, 7). Ferroptosis can be activated by some stimulator, including erastin, and is characterized by the accumulation of ROS and iron (8, 9). Ferroptosis is involved in the pathogenesis of various cancers (10), but the regulation mechanism of ferroptosis in thyroid cancer is elusive.

Circular RNAs are a new type of transcripts with the circular structure and are involved in gene regulation, chromatin modification, genome packaging, and genomic imprinting. Circular RNAs dysregulation is significantly correlated with abnormal cancer development. It has been reported that circular RNA DOCK1 down-regulates microRNA-124 and induces the growth of thyroid cancer cell proliferation (11). Circ-ABCB10 contributes to thyroid cancer cell invasion and proliferation through regulating KLF6 (12). Moreover, the expression of circ_0067934 is up-regulated in clinical thyroid cancer samples and the up-regulation of circ_0067934 is correlated with poor prognosis of thyroid cancer (13). Circ_0067934 enhances the malignant phenotypes by targeting Wnt/β-catenin and miR-1182/KLF8 signaling in non-small cell lung cancer (14). However, the impact of circ_0067934 on ferroptosis during thyroid cancer progression is still unreported. Previous studies have identified the critical role of microRNAs (miRNAs) in thyroid cancer. For instance, miR-145 is involved in lncRNA-TUG1-mediated progression of thyroid cancer (11). Furthermore, miR-545-3p has presented tumor suppressor function in various cancers, such as liver cancer, lung cancer, and ovarian cancer (15–17). But the function of miR-545-3p in thyroid cancer remains obscure.

In the present study, we identified a novel function of circ_0067934 in inhibiting ferroptosis and tumor growth of thyroid cancer by regulating miR-545-3p/SLC7A11.

## Materials and Methods

### Cell Culture

Normal thyroid follicular epithelial cell line Nthy-ori 3-1 and Human thyroid cancer cell lines FTC133 and TPC-1 cells were cultured in DMEM, comprising 10% FBS and 100U/ml penicillin/streptomycin at the humidified chamber of 37°C and 5% CO_2_. The shRNAs of circ_0067934 and SLC7A11 (LV-12 (pGLVH6-CMV-LUC-2A-Puro-U6-shRNA), pCMV-3tga-1A carrying SLC7A11 CDS (2μg) and circ_0067934 (2μg), miR-545-3p mimic (100 nM) and inhibitor (100 nM) were purchased (GenePharma/Genscript, China). The cells were treated with lentivirus at a multiplicity of infection (MOI) of 10 in the presence of Polybrene (8 ng/ml) or transfected with the specific plasmid by using Lipofectamine 3000 (ThermoFisher, USA, L3000001).

### Quantitative Reverse Transcription-PCR (RT-qPCR)

RNAs were isolated from tissues and cells by applying TRIZOL reagent (Biosntech, China, Lot B131905). Total RNAs were subjected into cDNA reverse transcription (Thermo, USA) and RT-qPCR assays were performed SYBR-Green (Vazyme, China, #P122). The data was normalized by GAPDH expression and the relative expression was calculated by 2^-ΔΔCt^.

The primer sequences:

circ_0067934 forward: 5′- TAGCAGTTCCCCAATCCTTG-3′

circ_0067934 reverse: 5′- CACAAATTCCCATCATTCCC-3′

miR-545-3p forward: 5′-TGGCTCAGTTCAGCAGGAAC-3′

miR-545-3p reverse: 5′-TGGTGTCGTGGAGTCG-3′

SLC7A11 forward: 5′-TGCTGGGCTGATTTTATCTTCG-3′

SLC7A11 reverse: 5′-GAAAGGGCAACCATGAAGAGG-3′

GAPDH forward: 5′- GAAGGTGAAGGTCGGAGTC-3′.

GAPDH reverse: 5′- GAAGATGGTGATGGGATTTC-3′

U6 forward: 5′-CTCGCTTCGGCAGCACA-3′

U6 reverse: 5′-AACGCTTCACGAATTTGCGT-3′

### MTT Assays

Cells were placed into 96-well plates for 24 hours after treatment, and were added with MTT reagent (Sigma, USA, M2128) for the incubation of 4 hours. The samples were treated with DMSO and observed in the POLARstar Omega system (BMG, Australia). The cells were treated with erastin (5 mmol/L, Sigma, USA, 571203-78-6) for the investigation of ferroptosis.

### Measurement Iron and ROS Levels

The iron and reactive oxygen species (ROS) levels were detected as previously reported (18, 19). The ROS levels were analyzed by DCFH-DA staining (Sigma, USA, 4091-99-0. 287810). The of iron and Fe^2+^ levels were measured by Iron Assay Kit (Sigma, USA, MAK025).

### Colony Formation Assays

For colony formation experiment, FTC133 and TPC-1 cells with the indicated treatment were placed in 6-well plate (1,000 cells per well), and incubated in complete medium for 2 weeks to form visible colonies. Subsequently, the colonies were washed, stained with crystal violet, and counted by a microscope (Carl Zeiss, German) and counted using ImageJ software.

### Analysis of Cell Apoptosis

The transfected FTC-133 and TPC-1 cells were digested and washed, suspended in 100 μL binding buffer and stained with Annexin V-FITC and PI staining reagents under the instruction of detection kit (CST, USA, #6592) in dark condition for 20 minutes. Afterward, the cells were washed and resuspended in binding buffer and measured by a flow cytometry (BD Biosciences, USA) immediately.

### Luciferase Reporter Gene Assay

To conduct dual-luciferase assay, cells were placed in a 24-well plate at a density of 5 × 10^4^ cells per well and transfected with wild type (WT) or mutated (MUT) luciferase reporter plasmids along with miR-186-5p mimics. After 48-hours transfection, luciferase reporter assay kit (Promega, USA, E1910) was adopted to measure luciferase activities. The WT and MUT plasmids containing the sequences of circ_0067934 and the 3’UTR of SLC7A11 were established by inserting the sequences into pmirGLO plasmids.

### Western Blot

Proteins with equal concentration were subjected into the sodium dodecyl sulfate–polyacrylamide gel electrophoresis (SDS-PAGE) and transferred in PVDF membrane (Bio-Rad, USA, 162-0177). The sample was blocked by non-fat milk (5%) and cultured by the antibodies of SLC7A11 (Abcam, USA, ab37185) and β-actin (Abcam, USA, ab8227) overnight at 4°C, followed by the secondary antibody incubation for 2 hours at 25°C. The expression was detected by ECL reagent (Sigma, USA, WBULS0100).

### Analysis of Tumorigenicity in Nude Mice

BALB/c nude mice (18-20 g, 4-week-old, male) (n = 6) were purchased and applied for the tumor growth analysis of FTC133 cells *in vivo*. About 1×106 FTC133 cells were transfected with control shRNA or circ_0067934 shRNA and were subcutaneously injected into the left fat pad of mice. After 5 days of injection, we measured tumor growth every 5 days. We sacrificed the mice after 30 days of injection. The tumor volume was calculated by (length × width^2^)/2. And the tumor weight was calculated at day 30. The experiments were approved by the Animal Ethics Committee of the First Affiliated Hospital of Harbin Medical University.

### Statistical Analysis

Data was quantified by means ± SD and compared using Student’s *t*-test or ANOVA (*P* < 0.05 as statistically significant) based on SPSS 16.0 software.

## Results

### Circ_0067934 Attenuates Ferroptosis of Thyroid Cancer Cells

**Figure 1:**
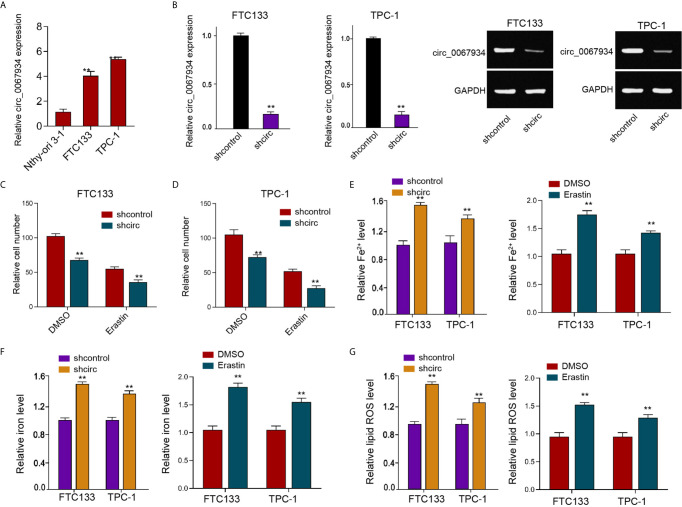
Circ_0067934 attenuates ferroptosis of thyroid cancer cells. **(A)** The analysis of circ_0067934 expression in Nthy-ori 3-1, FTC133 and TPC-1 cells. **(B)** The measurement of circ_0067934 expression in FTC133 and TPC-1 cells by RT-qPCR and RT-PCR. **(C, D)** The analysis of FTC133 and TPC-1 cell viability by MTT assays. **(E–G)** Analysis of Fe^2+^, iron, and ROS levels in FTC133 and TPC-1 cells. mean ± SD, **P < 0.01.

The expression of circ_0067934 was significantly enhanced in the FTC133 and TPC-1 cells compared with that in the normal thyroid follicular epithelial Nthy-ori 3-1 cells (**Figure 1A**). We validated that the circ_0067934 expression was depleted by shRNAs in FTC133 and TPC-1 cells (**Figure 1B**). The erastin reduced FTC133 and TPC-1 cell viabilities, while the circ_0067934 shRNA further repressed cell viabilities in erastin-treated FTC133 and TPC-1 cells (**Figures 1C, D**). The silencing of circ_0067934 increased the levels of ferroptosis-related markers, including Fe^2+^, iron, and ROS, in FTC133 and TPC-1 cells (**Figures 1E–G**), and the treatment of erastin served as a positive control, suggesting that circ_0067934 inhibits the thyroid cancer cell ferroptosis.

Moreover, the overexpression efficiency of circ_0067934 was validated in the FTC133 and TPC-1 cells (**Figure S1A**). The erastin repressed FTC133 and TPC-1 cell viabilities, while the circ_0067934 overexpression rescued cell viabilities in erastin-treated FTC133 and TPC-1 cells (**Figures S1B, C**). The overexpression of circ_0067934 reduced the levels of ferroptosis-related markers, including Fe^2+^, iron, and ROS, in FTC133 and TPC-1 cells (**Figures S1D–F**), and the treatment of erastin served as a positive control, confirming that circ_0067934 inhibits the thyroid cancer cell ferroptosis.

### Circ_0067934 Contributes to Cell Proliferation of Thyroid Cancer

**Figure 2:**
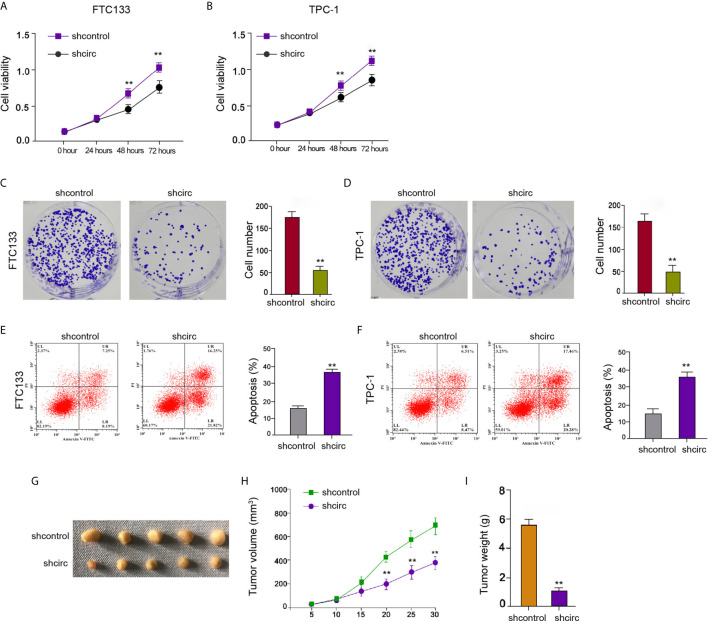
Circ_0067934 contributes to cell proliferation of thyroid cancer. **(A, B)** The analysis of FTC133 and TPC-1 cell viability by MTT assays. **(C, D)** Detection of cell proliferation of FTC133 and TPC-1 cells by colony formation assays. **(E, F)** Apoptosis analysis of FTC133 and TPC-1 cells by flow cytometry. **(G–I)** Tumor growth in nude mice by tumorigenicity assays. FTC133 cells were transfected with control shRNA or circ_0067934 shRNA and were subcutaneously injected into the left fat pad of mice. After 5 days of injection, we measured tumor growth every 5 days. We sacrificed the mice after 30 days of injection. The tumor size **(G)**, tumor volume **(H)**, and tumor weight **(I)** were demonstrated. mean ± SD, **P < 0.01.

We then observed that circ_0067934 depletion reduced FTC133 and TPC-1 cell viabilities (**Figures 2A, B**), implying that circ_0067934 contributes to the survival of thyroid cancer cells. The circ_0067934 silencing repressed FTC133 and TPC-1 cell colony formations as well (**Figures 2C, D**), indicating that circ_0067934 promotes the proliferation of thyroid cancer cells. The knockdown of circ_0067934 enhanced FTC133 and TPC-1 cell apoptosis *in vitro* (**Figures 2E, F**). Moreover, the inhibition of circ_0067934 by shRNA attenuated the growth of FTC133 cells *in vivo* (**Figures 2G–I**), suggesting that circ_0067934 enhances cell growth of thyroid cancer *in vivo*.

### Circ_0067934 Conversely Modulates miR-545-3p

**Figure 3:**
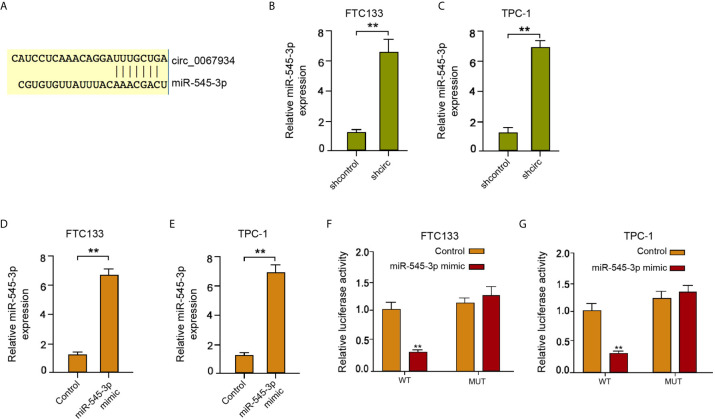
Circ_0067934 conversely modulates miR-545-3p. **(A)** The binding sites of circ_0067934 and miR-545-3p. **(B, C)** The measurement of miR-545-3p expression in FTC133 and TPC-1 cells by RT-qPCR. **(D–G)** The FTC133 and TPC-1 cells were treated with miR-545-3p mimic (100nM). **(D, E)** The detection of miR-545-3p expression in FTC133 and TPC-1 cells by RT-qPCR. **(F, G)** Measurement of luciferase activity of circ_0067934 by dual luciferase reporter assays in FTC133 and TPC-1 cells. mean ± SD, **P < 0.01.

In the circ_0067934-interacted miRNAs in bioinformatics analysis (CircInteractome online database), we found that miR-545-3p has presented tumor suppressor function in various cancers, such as liver cancer, lung cancer, and ovarian cancer (15–17), but the function of miR-545-3p has not been reported. Meanwhile, we identified that circ_0067934 inhibited ferroptosis of thyroid cancer cells and miR-545-3p potentially targeted ferroptosis-regulator SLC7A11 in a bioinformatics analysis (ENCORI online database). Consequently, we selected miR-545-3p for further investigation. The binding site of miR-545-3p with circ_0067934 was identified by bioinformatics analysis (CircInteractome online database) (**Figure 3A**). Circ_0067934 silencing enhanced miR-545-3p expression in FTC133 and TPC-1 cells (**Figures 3B, C**), implying that circ_0067934 may targeting miR-545-3p in thyroid cancer cells. The miR-545-3p mimic significantly enhanced miR-545-3p expression in FTC133 and TPC-1 cells (**Figures 3D, E**). The luciferase activity of circ_0067934, but not circ_0067934 containing miR-545-3p-binding site mutant, was attenuated by miR-545-3p mimic in FTC133 and TPC-1 cells (**Figures 3F, G**), suggesting that circ_0067934 serves as a sponge of miR-545-3p.

**Figure 4:**
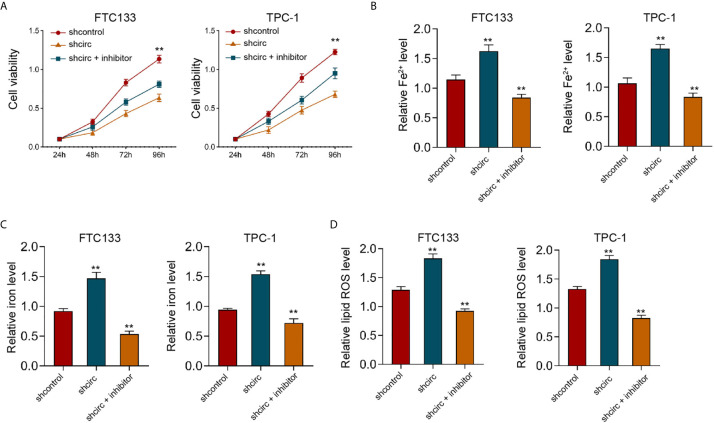
MiR-545-3p inhibitor reverses Circ_0067934 knockdown-enhanced ferroptosis of thyroid cancer cells. **(A)** The analysis of FTC133 and TPC-1 cell viability by MTT assays. **(B–D)** Analysis of Fe^2+^, iron, and ROS levels in FTC133 and TPC-1 cells. mean ± SD, **P < 0.01.

Moreover, our data showed that the depletion of circ_0067934 by shRNA repressed cell viabilities in erastin-treated FTC133 and TPC-1 cells, and miR-545-3p inhibitor could rescue the phenotype (**Figure 4A**). In addition, the levels of Fe^2+^, iron, and ROS were enhanced by circ_0067934 knockdown, while miR-545-3p inhibitor reversed this effect in FTC133 and TPC-1 cells (**Figures 4B–D**), indicating that circ_0067934 represses ferroptosis by targeting miR-545-3p.

### MiR-545-3p Negatively Targets SLC7A11

**Figure 5:**
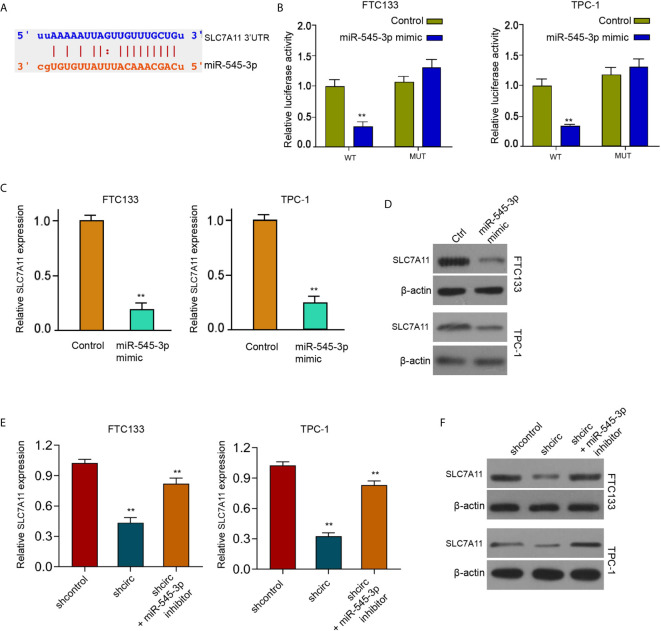
MiR-545-3p negatively targets SLC7A11. **(A)** The binding sites of SLC7A11 and miR-545-3p. **(B)** Measurement of luciferase activity of SLC7A11 by dual luciferase reporter assays in FTC133 and TPC-1 cells. **(C, D)** Detection of SLC7A11 expression in FTC133 and TPC-1 cells by RT-qPCR and Western blot analysis. **(E)** Analysis of SLC7A11 expression in FTC133 and TPC-1 cells by RT-qPCR **(F)** Western blot analysis of SLC7A11 expression in FTC133 and TPC-1 cells. mean ± SD, **P < 0.01.

We found that circ_0067934 inhibited ferroptosis of thyroid cancer cells and circ_0067934 sponged miR-545-3p in thyroid cancer cells. We also identified the miR-545-3p-targeted binding site of SLC7A11 by a bioinformatics analysis (ENCORI online database) (**Figure 5A**). The luciferase activity of SLC7A11, but not SLC7A11 containing miR-545-3p-binding site mutant, was repressed by miR-545-3p mimic in FTC133 and TPC-1 cells (**Figure 5B**), implying that miR-545-3p may affect mRNA 3’UTR of SLC7A11. The miR-545-3p mimic reduced SLC7A11 mRNA and protein levels in FTC133 and TPC-1 cells (**Figures 5C, D**), suggesting that miR-545-3p targets SLC7A11 in thyroid cancer cells. The silencing of circ_0067934 decreased SLC7A11 expression and miR-545-3p inhibitor rescued the expression in FTC133 and TPC-1 cells (**Figures 5E, F**), indicating that circ_0067934 up-regulating SLC7A11 by sponging miR-545-3p.

**Figure 6:**
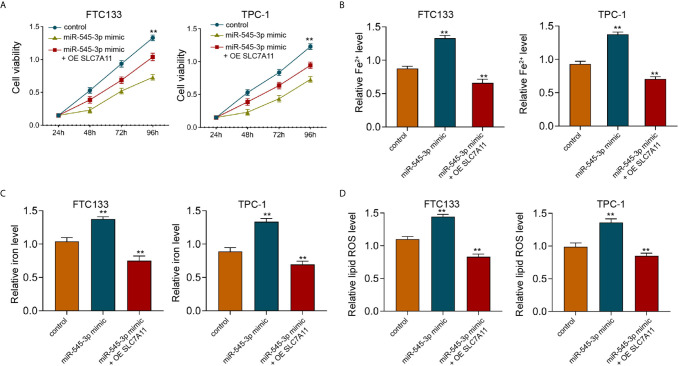
SLC7A11 overexpression reverses miR-545-3p -enhanced ferroptosis of thyroid cancer cells. **(A)** The analysis of FTC133 and TPC-1 cell viability by MTT assays. **(B–D)** Analysis of Fe^2+^, iron, and ROS levels in FTC133 and TPC-1 cells. mean ± SD, **P < 0.01.

Furthermore, we found that miR-545-3p mimic repressed cell viabilities in erastin-treated FTC133 and TPC-1 cells, and SLC7A11 overexpression could rescue the phenotype (**Figure 6A**). In addition, the levels of Fe^2+^, iron, and ROS were enhanced by miR-545-3p mimic, while SLC7A11 overexpression reversed this effect in FTC133 and TPC-1 cells (**Figures 6B–D**), suggesting that miR-545-3p contributes to ferroptosis of thyroid cancer cells by targeting SLC7A11.

**Figure 7:**
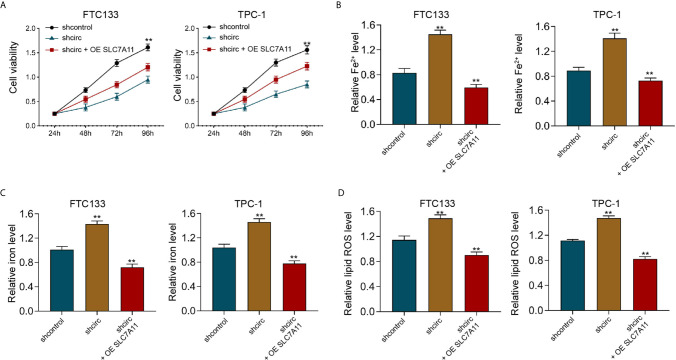
SLC7A11 overexpression reverses Circ_0067934 knockdown-enhanced ferroptosis of thyroid cancer cells. **(A)** The analysis of FTC133 and TPC-1 cell viability by MTT assays. **(B–D)** Analysis of Fe^2+^, iron, and ROS levels in FTC133 and TPC-1 cells. mean ± SD, **P < 0.01.

Meanwhile, the depletion of circ_0067934 by shRNA repressed cell viabilities in erastin-treated FTC133 and TPC-1 cells, and SLC7A11 overexpression could rescue the phenotype (**Figure 7A**). In addition, the levels of Fe^2+^, iron, and ROS were enhanced by circ_0067934 knockdown, while SLC7A11 overexpression reversed this effect in FTC133 and TPC-1 cells (**Figures 7B–D**), indicating that circ_0067934 represses ferroptosis of thyroid cancer cells by inducing SLC7A11.

### SLC7A11 Re-Constitution and miR-545-3p Inhibitor Reverse circ_0067934-Inhibited Thyroid Cancer Cell Growth

**Figure 8:**
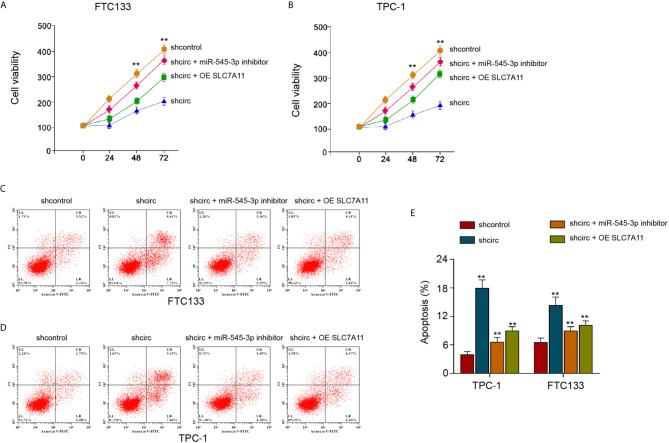
SLC7A11 re-constitution and miR-545-3p inhibitor reverse circ_0067934-inhibited thyroid cancer cell growth. **(A, B)** The analysis of FTC133 and TPC-1 cell viability by MTT assays. **(C–E)** Apoptosis analysis of FTC133 and TPC-1 cells by flow cytometry. mean ± SD, **P < 0.01.

Next, we found that the silencing of circ_0067934 attenuated FTC133 and TPC-1 cell viabilities, and miR-545-3p inhibitor or SLC7A11 overexpression was able to rescue the cell viabilities (**Figures 8A, B**). The FTC133 and TPC-1 cell apoptosis was enhanced by circ_0067934 depletion, while miR-545-3p inhibitor or SLC7A11 overexpression could block this phenotype in the cells (**Figures 8C–E**). Together these data imply that circ_0067934 regulates cell growth and ferroptosis of thyroid cancer cells by miR-545-3p/SLC7A11 axis.

## Discussion

Thyroid cancer is a prevailing endocrine malignancy in the world with high mobility and severe morbidity. The ferroptosis, as an emerging cellular process and the critical therapeutic target, plays essential roles in the development of cancer. However, the investigation of ferroptosis in the thyroid cancer progression is extremely limited. The mechanisms of ferroptosis are complicated during cancer development. In this study, we identified that circ_0067934 attenuated ferroptosis of thyroid cancer cells. Our finding provides valuable evidence and enriches understanding of the role of ferroptosis in the pathogenesis of thyroid cancer. These data not only present a novel function of circ_0067934 in regulating ferroptosis but also improved the understanding of the regulatory mechanisms of ferroptosis in thyroid cancer development. Moreover, circular RNAs are reported to regulate the progression of thyroid cancer by various mechanisms. It has been reported that Circ_102171 enhances tumorigenesis by regulating CTNNBIP/β-catenin signaling in thyroid cancer (20). Circ_0058124 enhances the progression of thyroid cancer *via* the NOTCH3/GATAD2A signaling (21). CircFOXM1 contributes thyroid cancer through miR-1179/HMGB1 signaling (22). CircRNA_NEK6 modulates thyroid cancer development by miR-370-3p/Wnt signaling (23). CircTP53 enhances tumor growth by regulating miR-1233-3p/MDM2 signaling in thyroid cancer (24). Meanwhile, the expression of circ_0067934 is elevated in clinical thyroid cancer tissues and the elevation of circ_0067934 is associated with poor prognosis of thyroid cancer (13), but the effect of circ_0067934 on thyroid cancer progression is still unreported. In this study, our data identified that Circ_0067934 contributed to proliferation of thyroid cancer cells. We elucidate the critical role of Circ_0067934 in regulating thyroid cancer progression in both cell model and mice model. Here, we just investigated the function of Circ_0067934 in thyroid cancer at the experimental conditions and the therapeutic and clinical value of Circ_0067934 in thyroid cancer are needed to explore in the future. Moreover, apoptosis and ferroptosis are both crucial phenotypes during cancer development. As several previous reports (19, 25–27), silencing the circ_0067934 not only induces cell ferroptosis but also causes cell apoptosis. The authors should distinguish which is the main cause of the observed cell death in future investigations, despite it is difficult and complicated.

MiRNAs are the critical downstream regulators of circular RNAs-mediated cancer development. It has been reported that miR−148a reduces thyroid cancer cell proliferation by PI3K/AKT and STAT3 signaling (28). MiR-335-5p/ICAM-1 axis represses thyroid cancer metastasis (29). MiR-539 suppresses invasion and migration by targeting CARMA1 in thyroid cancer (30). MiR-592 decreases thyroid cancer progression by modulating lncRNA NEAT1/NOVA1 signaling (31). MiR-650 enhances thyroid cancer cell motility by regulating PPP2CA (32). Furthermore, it has been found that up-regulation of SLC7A11 is correlated with poor prognosis of thyroid cancer (33). We found that Circ_0067934 up-regulated SLC7A11 by down-regulating miR-545-3p and miR-545-3p inhibitor and SLC7A11 overexpression could reverse the cell proliferation regulated by Circ_0067934 depletion in thyroid cancer cells. These data indicate an unreported mechanism of Circ_0067934, miR-545-3p, and SLC7A11 in the modulation of thyroid cancer progression. MiR-545-3p/SLC7A11 axis may just one of the downstream mechanisms of Circ_0067934-regulated thyroid cancer development and other mechanisms deserve to investigate in future studies. Importantly, SLC7A11 has emerged as a central hub linking ferroptosis to its proposed tumor suppression function. SLC7A11-mediated ferroptosis inhibition not only plays a role in tumor development caused by loss of tumor suppressors (such as p53 and BAP1) but also contributes to oncogene-driven tumorigenesis (34). In this study, we identified that miR-545-3p targets and repressed SLC7A11 expression in thyroid cancer cells. The overexpression of SLC7A11 could attenuate miR-545-3p-induced ferroptosis of thyroid cancer cells. The correlation and effect of miR-545-3p and SLC7A11 in the modulation of tumorigenesis and malignant progression of thyroid cancer cells should be explored in more investigations.

Therefore, we concluded that Circ_0067934 attenuated ferroptosis of thyroid cancer cells by miR-545-3p/SLC7A11 signaling. Circ_0067934 may serve as a potential therapeutic target by regulating ferroptosis for the treatment of thyroid cancer.

## Data Availability Statement

The original contributions presented in the study are included in the article/**Supplementary Material**. Further inquiries can be directed to the corresponding author.

## Ethics Statement

The animal study was reviewed and approved by First Affiliated Hospital of Harbin Medical University.

## Author Contributions

HW and JM designed and performed experiments, analyzed data, and wrote the paper. XZ designed and performed experiments. JM designed experiments, analyzed data, and wrote the paper. All authors contributed to the article and approved the submitted version.

## Funding

This study was financial supported by Project of Health Commission of Heilongjiang Province and project number: 2020-425.

## Conflict of Interest

The authors declare that the research was conducted in the absence of any commercial or financial relationships that could be construed as a potential conflict of interest.

## Supplementary Material

Supplementary Figure 1 Circ_0067934 attenuates ferroptosis of thyroid cancer cells. **(A)** The measurement of circ_0067934 expression in FTC133 and TPC-1 cells by RT-qPCR and RT-PCR. **(B, C)** The analysis of FTC133 and TPC-1 cell viability by MTT assays. **(D–F)** Analysis of Fe^2+^ **(D)**, iron **(E)**, and ROS **(F)** levels in FTC133 and TPC-1 cells. mean ± SD, ***P* < 0.01.
