# Supplementary figures and images for: Circular RNA Circ_0067934 Attenuates Ferroptosis of Thyroid Cancer Cells by miR-545-3p/SLC7A11 Signaling
Source: Front Endocrinol (Lausanne). 2021 Jul 5;12:670031. doi: 10.3389/fendo.2021.670031 (PMC8287831; doi:10.3389/fendo.2021.670031)

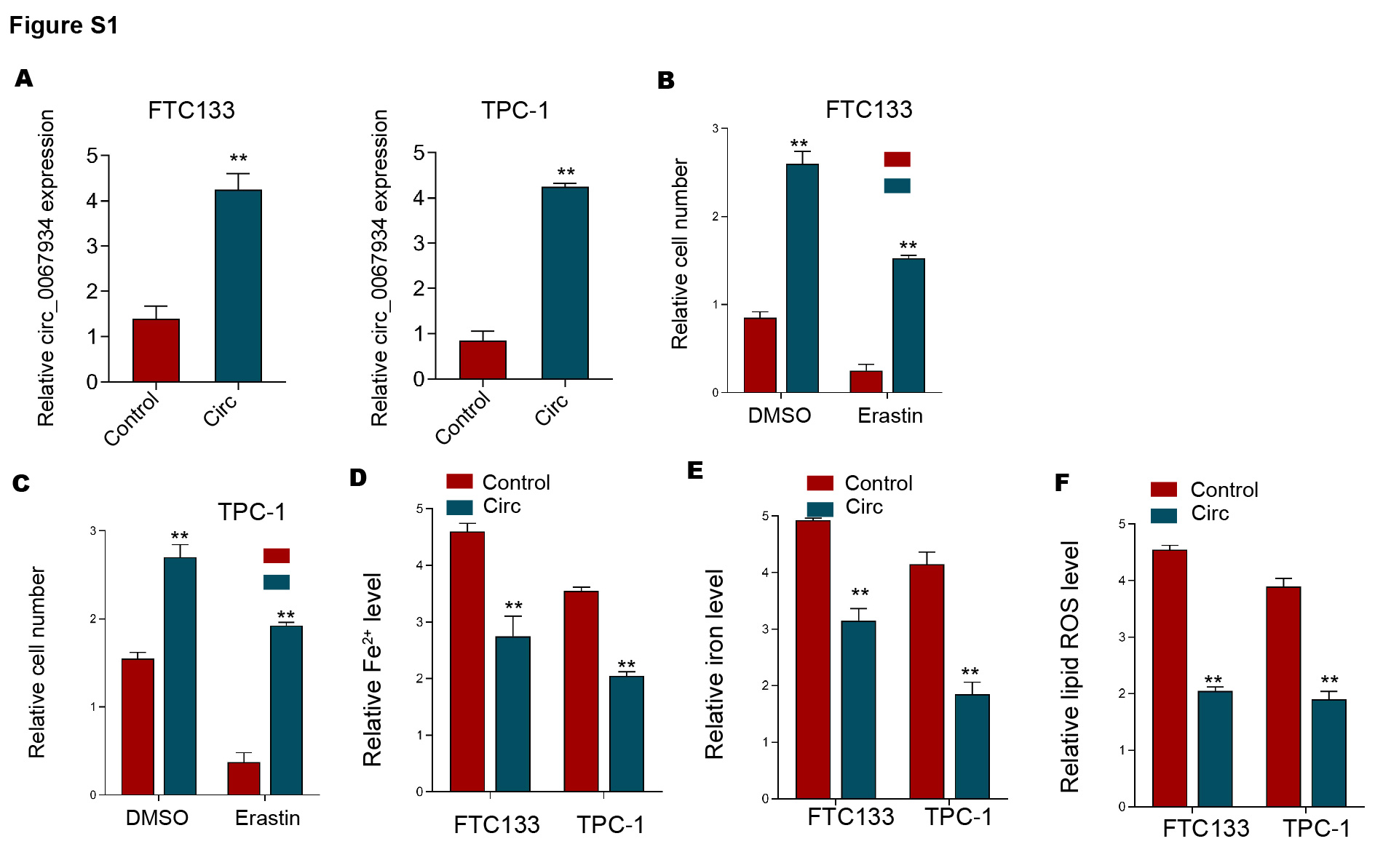

Supplement: Supplementary Figure 1 — Circ_0067934 attenuates ferroptosis of thyroid cancer cells. (A) The measurement of circ_0067934 expression in FTC133 and TPC-1 cells by RT-qPCR and RT-PCR. (B, C) The analysis of FTC133 and TPC-1 cell viability by MTT assays. (D–F) Analysis of Fe2+ (D), iron (E), and ROS (F) levels in FTC133 and TPC-1 cells. mean ± SD, **P < 0.01. [file Image_1.jpeg]
